# Association analysis of juvenile idiopathic arthritis genetic susceptibility factors in Estonian patients

**DOI:** 10.1007/s10067-021-05756-x

**Published:** 2021-06-08

**Authors:** Tiit Nikopensius, Priit Niibo, Toomas Haller, Triin Jagomägi, Ülle Voog-Oras, Neeme Tõnisson, Andres Metspalu, Mare Saag, Chris Pruunsild

**Affiliations:** 1 Estonian Genome Center, Institute of Genomics, University of Tartu, Riia 23B, 51010 Tartu, Estonia; 2 Institute of Dentistry, University of Tartu, Tartu, Estonia; 3 Stomatology Clinic, Tartu University Hospital, Tartu, Estonia; 4 Department of Clinical Genetics, United Laboratories, Tartu University Hospital, Tartu, Estonia; 5 Children’s Clinic, Tartu University Hospital, Tartu, Estonia; 6 Children’s Clinic, Institute of Clinical Medicine, Faculty of Medicine, University of Tartu, Tartu, Estonia

**Keywords:** Genome-wide association study, Juvenile idiopathic arthritis, Rheumatoid arthritis

## Abstract

**Background:**

Juvenile idiopathic arthritis (JIA) is the most common chronic rheumatic condition of childhood. Genetic association studies have revealed several JIA susceptibility loci with the strongest effect size observed in the human leukocyte antigen (HLA) region. Genome-wide association studies have augmented the number of JIA-associated loci, particularly for non-HLA genes. The aim of this study was to identify new associations at non-HLA loci predisposing to the risk of JIA development in Estonian patients.

**Methods:**

We performed genome-wide association analyses in an entire JIA case–control sample (All-JIA) and in a case–control sample for oligoarticular JIA, the most prevalent JIA subtype. The entire cohort was genotyped using the Illumina HumanOmniExpress BeadChip arrays. After imputation, 16,583,468 variants were analyzed in 263 cases and 6956 controls.

**Results:**

We demonstrated nominal evidence of association for 12 novel non-HLA loci not previously implicated in JIA predisposition. We replicated known JIA associations in *CLEC16A* and *VCTN1* regions in the oligoarticular JIA sample. The strongest associations in the All-JIA analysis were identified at *PRKG1* (*P* = 2,54 × 10^−6^), *LTBP1* (*P* = 9,45 × 10^−6^), and *ELMO1* (*P* = 1,05 × 10^−5^). In the oligoarticular JIA analysis, the strongest associations were identified at *NFIA* (*P* = 5,05 × 10^−6^), *LTBP1* (*P* = 9,95 × 10^−6^), *MX1* (*P* = 1,65 × 10^−5^), and *CD200R1* (*P* = 2,59 × 10^−5^).

**Conclusion:**

This study increases the number of known JIA risk loci and provides additional evidence for the existence of overlapping genetic risk loci between JIA and other autoimmune diseases, particularly rheumatoid arthritis. The reported loci are involved in molecular pathways of immunological relevance and likely represent genomic regions that confer susceptibility to JIA in Estonian patients.

**Table:** 

**Key Points** • *Juvenile idiopathic arthritis (JIA) is the most common childhood rheumatic disease with heterogeneous presentation and genetic predisposition.* • *Present genome-wide association study for Estonian JIA patients is first of its kind in Northern and Northeastern Europe.* • *The results of the present study increase the knowledge about JIA risk loci replicating some previously described associations, so adding weight to their relevance and describing novel loci.* • *The study provides additional evidence for the existence of overlapping genetic risk loci between JIA and other autoimmune diseases, particularly rheumatoid arthritis.*

**Supplementary Information:**

The online version contains supplementary material available at 10.1007/s10067-021-05756-x.

## Background

Juvenile idiopathic arthritis (JIA) is the most common chronic rheumatic disease of childhood, encompassing all forms of chronic inflammatory arthritis of unknown cause and having an onset before the age of 16 years. JIA can be subdivided into seven distinctive categories according to the International League of Associations for Rheumatology (ILAR) classification criteria [1]. Prevalence and incidence studies have outlined a great extent of ethnic diversity within JIA; however, JIA is more common in populations of European ancestry, particularly in Northern European populations with the highest prevalence varying around 1/1,000 [2, 3]. As JIA is much less common than adult-onset rheumatoid arthritis (RA), the assembly of large samples required for genetic studies is challenging.

There is a strong genetic contribution to the risk of JIA, and multiple susceptibility loci have been established through genome-wide association studies [GWASs) of the most common JIA subtypes oligoarticular and rheumatoid factor (RF)-negative polyarticular JIA, with 17 genome-wide and 20 suggestively significant associations described [4, 5]. The human leukocyte antigen (HLA) region and the top 27 non-HLA loci were estimated to explain 13% and 6%, respectively, of the risk of JIA [4]. Many identified JIA loci have not been characterized functionally, as the majority of associated variants is in intronic and intergenic regions, with their presumable functions accented on target gene expression regulation and not directly affecting the sequence of any protein product [4].

The prevalence of other autoimmune diseases is increased in the relatives of patients with JIA, and particularly for RA – sharing the most similar clinical and pathological features with JIA, and considerable overlap in genetic susceptibility loci for these two traits has been outlined [5, 6]. Many additional RA loci have been identified in recent years, and they likewise may represent acceptable candidate loci for JIA. As a significant proportion of JIA heritability risk remains unclear, it is important to conduct comparative studies in different populations, considering that the replication of previous findings is paramount to determine true risk loci, thus unraveling key disease-related pathways warranting further in-depth studies.

In this study, we adopted a GWAS approach to detect novel loci associated with JIA and replicate several previously reported signals of association, particularly in non-HLA regions in an Estonian JIA case–control sample. We also sought to ascertain additional associations of common variants with lower odds ratios (ORs), as well as uncommon risk alleles with higher odds ratios (ORs).

## Methods

### Study population

The case group consisted of 263 consecutive Estonian patients with JIA (146 girls and 117 boys) who fulfilled the ILAR diagnostic criteria and had JIA onset at < 16 years of age. Patients were subsequently included in the study during regular visits to the pediatric rheumatologist. The mean age at onset of JIA for the whole sample was 10 years and 5 months (IQR 5 − 13 years); 8 years and 4 months (IQR 4 − 12 years) for boys; and 11 years (IQR 6 − 13 years 5 months) for girls, respectively.

The distribution of JIA subtypes reflected typical proportions, comprising mainly oligoarthritis (86 persistent and 109 extended) and polyarthritis (56 rheumatoid factor (RF)-negative and 2 RF-positive); four patients had psoriatic arthritis, three had enthesitis-related arthritis, and one patient of each had systemic and other arthritis. Additional descriptive data are presented in Supplementary Table S1.

Six thousand ninety hundred and fifty-six healthy controls were selected from the Estonian Biobank cohort. All controls lacked medical records of RA (neither seropositive (M05) nor seronegative (M06)), juvenile arthritides (and with other diseases, M08 and M09), seronegative spondyloarthropathies (M45), and non-specified arthritides (M13). Individuals with family history of JIA or RA were excluded.

### Ethics statement

Ethical approval for this study was obtained from the Research Ethics Review Committee of the University of Tartu. All patients and controls provided informed consent for participation in the study.

### Genotyping

For the GWAS, all discovery samples were genotyped on the Illumina HumanOmniExpress BeadChip microarrays according to the manufacturer’s protocol. After pruning of single-nucleotide polymorphisms (SNPs) in linkage disequilibrium (*r*^2^ > 0.2), EIGENSTRAT was used to compute principal components for use as covariates in the regression analyses. Principal-component analysis (PCA) was performed using EIGENSTRAT to identify ancestry outliers.

Quality control procedures included the following exclusion criteria: marker call rate < 0.95, minor allele frequency (MAF) < 0.01, sample call rate (missingness) < 0.95, and significant departure from Hardy–Weinberg equilibrium (*P* < 1 × 10^−6^). We excluded population outliers and all individuals who had unidentifiable sex discrepancies.

### Imputation

For imputation, we obtained high-coverage (~ × 40) whole-genome sequencing data from 2240 population-based controls from Estonian Biobank. After quality control, we constructed a reference panel including 16,583,468 single-nucleotide variants (SNVs). The imputation of this nationwide reference panel substantially increases the imputation accuracy for low-frequency variants (allele frequency 0.5 − 10%) in comparison to commonly used reference panels such as 1000 Genomes Project Phase 3. Imputation was performed using IMPUTE version 2.2.2.

### Statistical analysis

Variants were tested for an association with JIA case–control status by logistic regression. Primary inference was based on an additive genetic model. Case–control association tests were performed with SNPTEST version 2.5.2, applying gender and first four PCA axes as covariates. Considering our available sample size, we selected *P* < 5 × 10^−8^ as the threshold for genome-wide significance; *P* < 1 × 10^−4^ level was considered to represent nominal significance for associations. The genomic control inflation factor value was calculated for the whole dataset, and showed only very modest inflation (λ_GC_ = 1.04), indicating adequate control of population stratification. Only genotyped SNPs of high quality were used to inform imputation. Imputed SNP quality was assessed using the information score (> 0.6) and the confidence score (> 0.9).

## Results

In total, 16,5 million SNVs were genotyped in the Estonian sample. Table 1 presents the strongest associations (*P* < 1 × 10^−4^) in the entire case–control sample (All-JIA) and Table 2 in an oligoarticular JIA endophenotype (OA-JIA) sample.

**Table 1:** Nominally significant case–control association test results: All-JIA sample

Chr	Position	dbSNP ID	Alleles	Gene (region)	MAF		*P*	OR	95% CI
			(Ref/Alt)						
					Cases	Controls			
1	18,013,430	rs12742364	T/A	*PADI4*	0,096	0,154	5,06 × 10^−5^	0,583	0,434–0,782
2	33,218,037	rs219158	A/G	*LTBP1*	0,513	0,413	9,45 × 10^−6^	1,498	1,258–1,783
6	106,629,698	rs182399829	A/T	*PRDM1*	0,400	0,327	7,23 × 10^−5^	1,371	1,148–1,638
6	106,651,006	rs73776556	C/T	*ATG5*	0,389	0,315	3,20 × 10^−5^	1,389	1,161–1,661
7	37,157,641	rs2111035	C/A	*ELMO1*	0,245	0,160	1,05 × 10^−5^	1,703	1,389–2,087
8	122,539,123	rs1564569	A/G	*HAS2*	0,337	0,251	2,47 × 10^−5^	1,510	1,255–1,817
10	53,762,921	rs2454539	C/T	*PRKG1*	0,513	0,413	2,54 × 10^−6^	1,501	1,261–1,787
22	24,420,128	rs9624387	C/T	*CABIN1*	0,062	0,031	3,74 × 10^−5^	2,066	1,429–2,985

*Chr* chromosome; *MAF* minor allele frequency; *OR* odds ratio; *CI* confidence intervals

**Table 2:** Nominally significant case–control association test results: OA-JIA sample

Chr	Position	dbSNP ID	Alleles	Gene (region)	MAF		*P*	OR	95% CI
			(Ref/Alt)						
					Cases	Controls			
1	62,090,537	rs1436238830	T/A	*NFIA*	0,478	0,377	5,05 × 10^−6^	0,661	0,540–0,809
1	117,884,022	rs11586858	G/A	*VTCN1*	0,178	0,256	5,80 × 10^−5^	1,590	1,222–2,067
2	33,218,037	rs219158	A/G	*LTBP1*	0,528	0,413	9,95 × 10^−6^	1,591	1,300–1,947
3	112,484,400	rs62263666	T/C	*CD200R1*	0,265	0,347	2,59 × 10^−5^	0,677	0,539–0,850
7	37,157,641	rs2111035	C/A	*ELMO1*	0,247	0,160	9,37 × 10^−5^	1,723	1,362–2,179
10	61,620,478	rs141412077	A/G	*UBE2D1*	0,076	0,034	1,45 × 10^−5^	2,357	1,600–3,473
13	81,150,170	rs2154130	C/T	*SPRY2*	0,322	0,433	6,18 × 10^−5^	1,605	1,294–1,991
16	11,563,226	rs11074991	C/A	*CLEC16A*	0,204	0,292	6,03 × 10^−5^	0,620	0,483–0,796
19	6,785,961	rs808843	G/A	*VAV1*	0,159	0,103	2,75 × 10^−5^	1,637	1,240–2,160
21	42,816,368	rs28449257	C/G	*MX1*	0,250	0,324	1,65 × 10^−5^	0,696	0,551–0,878

*Chr* chromosome; *MAF* minor allele frequency; *OR* odds ratio; *CI* confidence intervals

In total, 1131 SNPs showed nominal evidence for association in the All-JIA analysis. Seven biologically plausible new JIA loci had nominally significance associations with variants in *PADI4, LTBP1*, *PRDM1*/*ATG5*, *ELMO1*, *HAS*2, *PRKG1*, and *CABIN1* defined as genomic regions not previously associated with the trait. None of the markers reached the genome-wide level of significance. The most strongly associated variants were identified at *PRKG1* rs2454539 (*P* = 2,54 × 10^−6^), *LTBP1* rs219158 (*P* = 9,45 × 10^−6^), and *ELMO1* rs2111035 (*P* = 1,05 × 10^−5^). All top variants, except for *PADI4*, were associated with a higher risk of JIA development.

In total, 1067 SNPs showed nominal evidence for association in the OA-JIA analysis. We replicated previously described association signals [7] for *CLEC16A* region with the top variant rs11074991 (*P* = 6,03 × 10^−5^) and *VCTN1* [8] with the top variant rs11586858 (*P* = 5,80 × 10^−5^). Seven biologically plausible new loci showed nominally significant associations with variants in regions neighboring *NFIA*, *LTBP1, CD200R1*, *ELMO1*, *SPRY2*, *VAV1*, and *MX1*. The most strongly associated variants were identified at *NFIA* rs1436238830 (*P* = 5,05 × 10^−6^), *LTBP1* rs219158 (*P* = 9,95 × 10^−6^), *MX1* rs28449257 (*P* = 1,65 × 10^−5^), and *CD200R1* rs62263666 (*P* = 2,59 × 10^−5^). Top variants in *LTBP1*, *NFIA*, *ELMO1*, *UBE2D1*, and *VAV1* were associated with a higher risk of oligoarticular JIA development.

The above-mentioned genes have roles in immune system regulation and/or major histocompatibility complex (MHC) antigen processing and presentation, apoptosis, and T cell receptor repertoire. Some genes are associated with other autoimmune diseases.

## Discussion

This paper describes a genome-wide association (GWA) study to identify novel JIA susceptibility loci outside HLA region, performed in an Estonian JIA case–control sample (263 cases and 6956 controls). Variants were tested for association with JIA risk by logistic regression. The majority of reported associations appeared in genes encoding proteins that have roles in immune system regulation and/or MHC antigen processing and presentation. As expected, most associated regions fall within non-coding regulatory regions. The majority of reported nominally significant associations overlap with previously reported susceptibility loci for RA; overlaps with other autoimmune diseases were observed for *UBE2D1* associated with Crohn’s disease [9], *PRDM1* with systemic sclerosis [10], and *VAV1* with multiple sclerosis [11]. Altogether, we identified 12 novel loci not previously implicated in genetics of JIA and/or JIA subtypes.

We have replicated previously described association of the *CLEC16A* gene region with the risk for JIA and also anti-cyclic citrullinated peptide antibody [anti-CCP] negative RA in Norwegian Caucasians [7]. We report association in oligoarticular JIA sample. GWASs have also shown that *CLEC16A* variants confer susceptibility to autoimmune diseases such as type 1 diabetes and multiple sclerosis; with evidence of association with RA [12]. *CLEC16A* – C-type lectin domain containing 16A – is expressed almost exclusively in immune cells, such as CD4 and CD8 T cells, dendritic cells, B lymphocytes, and natural killer (NK) cells, demonstrating its importance in immune system regulation. Our findings provide further support for *CLEC16A* as an autoimmune risk locus, and particularly for oligoarticular JIA. Further efforts are necessary to elucidate the function of *CLEC16A*, and to identify actual causal variants, which appear to be distinct for different autoimmune diseases.

The second replicated JIA locus, for which we report an association in OA-JIA sample, is *VTCN1* – V-set domain-containing T cell activation inhibitor 1 – showing strongest association with JIA among non-HLA genes in previous studies [13, 14], and has been implicated as a potentially predictive disease-course marker for selected JIA subtypes [8].

The *ATG5/PRDM1* region has been linked to susceptibility of several autoimmune diseases, including RA [15, 16] and systemic lupus erythematosus (SLE) [17]. *ATG5* (autophagy-related 5)-encoded protein is involved in MHC class II antigen presentation, apoptosis, and lymphocyte development and is essential for B and T lymphocyte survival and proliferation. *PRDM1* – PR/SET domain 1 – encodes a transcription factor that mediates a transcriptional program in various innate and adaptive immune tissue-resident lymphocyte T cell types. Increased mRNA and protein expression levels of autophagy-related proteins, including Atg5, were found in the synovial tissue of RA patients [18]. The inhibition of autophagy and increased apoptotic activation correlates with a favorable clinical outcome in RA patients treated with anti-tumor necrosis factor (TNF) drugs [19]. This finding suggests that reducing the expression levels of autophagy-related genes might become a new therapeutic target for active RA; the *ATG5* suppressor oridonin was recently introduced as a potential therapeutic target for RA [20].

Silencing of transcription factor Prdm1 (B lymphocyte-induced maturation protein-1; Blimp1) in osteoclast precursor cells attenuated the stimulatory effect of TNF-α on osteoclastogenesis; however, TNF-α-induced Prdm1 expression was rescued discernibly by blocking the PI3K/AKT signaling pathway. Upregulation of Prdm1 expression by TNF-α through the activation of PI3K/AKT signaling during osteoclastogenesis seems a plausible molecular mechanism regulating TNF-α-induced osteoclasts differentiation that has profound effects in RA [21]. Interleukin-23 (IL-23) is a pro-inflammatory cytokine required for the pathogenicity of T helper 17 (Th17) cells, and Prdm1 was identified as a key IL-23-induced factor that activates the Th17 inflammatory program [22]. Interactions between Th17 cells and their effector molecules interferon (IFN)-γ and TNF-α are implicated in the pathology of RA, and *PRDM1* – among other established genetic risk factors for RA – is directly involved in Th17 cell differentiation and/or function. Interactions between Th17 cells and other immune cells in the synovial tissue during the early phases of RA lead to chronic inflammation, irreversible cartilage degradation, and bone erosion [23]. Downregulation of the expression of Prdm1, a repressor for negative regulators of osteoclast differentiation Irf8 and Bcl6, was proposed as another mechanism of action for compounds possessing effective therapeutic potential for the treatment and/or prevention of bone loss [24].

*LTBP1* – latent-transforming growth factor beta-binding protein 1 – encodes a component of the large latent TGF-β complex binding TGF-β to the extracellular matrix and acts as key regulator of latent-state TGF-β activation; overexpression of TGF-β-related genes supports the importance of this pathway in synovial pathology. Global gene expression profiling of JIA fibroblast-like synoviocytes (FLS) exhibited a hypertrophic chondrocyte phenotype and demonstrated that dysregulation of TGF-β signaling pathway along with contributions from the upregulated β-catenin signaling pathway, leading to inhibition of chondrocyte differentiation, and partially downregulated canonical Wnt signaling pathway may have implications for endochondral bone formation and contribute to bony overgrowth in oligoarticular JIA. Moreover, overexpression of bone morphogenetic protein 4 (BMP4) in FLSs from patients with oligoarticular JIA was inferred to have a direct effect on functional outcome in terms of disease pathogenesis and was implicated as a target for future treatment [25]. Recently, involvement of the TGF-β1/Smad signaling pathway in epithelial-mesenchymal transition and contribution to migration and invasion in RA FLSs were shown [26], warranting further studies of JIA FLSs.

*ELMO1* (engulfment and cell motility 1)-encoded protein is involved in cytoskeletal rearrangements required for phagocytosis of apoptotic cells and cell migration. Integrative omics analysis demonstrated enhanced expression of ELMO1 protein and hypomethylation of *ELMO1* locus, ELMO1-promoted cell migration and invasion, and Rac1 activity regulation in RA FLSs, implicating a link between *ELMO1* and RA pathogenicity and the potential of *ELMO1* as a RA therapeutic target [27]. Additionally, loss of the apoptotic cell-engulfment signaling protein ELMO1 alleviated disease severity in mouse models of arthritis through regulation of neutrophil chemotaxis to inflamed joints, and *ELMO1* knockdown reduces human neutrophil migration to chemokines linked to inflammatory arthritis [28].

AKT and ERK pathways are known to be activated in human RA FLSs, which play crucial roles in RA pathogenesis and joint destruction. *SPRY2* – sprouty RTK signaling antagonist 2 – has been known as a tumor suppressor by preventing ERK and AKT signaling activation. By downregulation of Ras/Raf/ERK and PTEN/PI3K/AKT signaling, Spry2 overexpression suppressed AKT and ERK pathways and production of proinflammatory cytokines IL-1β, IL-6, matrix metalloproteinase (MMP)-1, and MMP-3 and effectively inhibited the hyperproliferation induced by TNF-α in RA FLSs [29]. Thus, *SPRY2* has a significantly suppressive effect on inflammatory responses in RA, and the enhancement of SPRY2 activity in FLSs contributes to the amelioration or prevention-orientated treatment of RA.

*NFIA* encodes a member of the NF1 (nuclear factor 1) family of transcription factors and is expressed in CD8 T cells. *NFIA* has been shown to regulate the production, differentiation, and/or function of immune cell subsets of the innate immune system, including monocytes/macrophages [30] and CD314 − CD158a + NK cells [31]. *NFIA* represents an example of genetic susceptibility factors specific for anti-CCP-negative RA, with the presumption that production of anti-CCP antibodies additionally requires the engagement of the adaptive immune system [32].

*VAV1* – Vav guanine nucleotide exchange factor 1 – encoded protein is important in hematopoiesis, participating in T and B cell development and activation. *VAV1* variants were also associated with anti-CCP negative RA, with an involvement in modulation of T cell signaling [33].

Experimental studies have revealed that CD200/CD200R1 signaling pathway has an immunosuppressive effect on the inflammatory cellular immune response and maintains immune homeostasis in the context of autoimmune diseases. Significant negative correlation between *CD200R1* – CD200 receptor 1 – expression in monocytes-derived macrophages and disease activity in RA patients was shown to support potential involvement of the CD200/CD200R1 pathway in the pathogenesis of RA and implicate *CD200R1* as a valuable biomarker of disease activity [34]. Aberrant CD200/CD200R1 expression in RA was shown to contribute to abnormal Th17 cell differentiation, chemotaxis, and osteoclastogenesis; this abnormal expression was corrected after treatment with a TNF-α antagonist plus methotrexate, demonstrating that CD200/CD200R1 exerts anti-inflammatory functions via multiple mechanisms and delineating a potential immunotherapeutic role for targeting CD200/CD200R1 signaling in RA [35].

Numerous association studies focusing on *PADI4* – peptidyl arginine deiminase 4 – variants and RA risk in different populations have given controversial results in different populations. Recent meta-analyses have revealed diversity among different *PADI4* variants contributing to RA susceptibility in Asian, but not European populations, as well as in both populations [36–38]. The involvement of *PADI4* rs2240340 variant in the risk of RA development in patients positive for autoantibodies to citrullinated antigens was shown only for HLA-DRB1*04 non-carriers [39].

In contrast to a previous report where no associations were found between JIA clinical subtypes and *PADI4* variants [40], we demonstrated an association with overall JIA in Estonian population. An anti-apoptotic role of *PADI4* in RA development was proposed as a molecular mechanism contributing to RA pathogenesis as the knockdown of *PADI4* promoted the apoptosis of FLSs and upregulated the expression of p53 and p21 [41].

*PRKG1* – protein kinase CGMP-dependent 1 – has demonstrated the strongest association with high levels of circulating IFN-α in SLE patients [42]. The biological function for *PRKG1* in type I IFN production or signaling was supported in dendritic cells and NK cells, which conjointly generate IFN-α in SLE [43]; however, *PRKG1* function was less strongly supported in T and B lymphocytes, which are not considered to be major IFN-α producing cells [42].

*MX1* – MX dynamin-like GTPase 1 – is known as an important contributor in the type 1 interferon pathway, and the type I IFN signature in RA has shown clinical relevance in relation to disease onset and therapeutic response. Increased *MX1* expression levels were shown to be correlated with disease activity in fibroblast cells of synovial tissue in RA patients with high C-reactive protein levels [44]. Gene expression signatures in RA synovial samples have shown downregulation of the protein–protein interaction network comprising *MX1* and significant enrichment of this network in the immune response pathway [45]. A recent study demonstrated that RA-associated DNA methylation sites have regulatory effects on mRNA expressions and create an IFN-inducible gene interaction network (comprising *MX1*) associated with RA [46].

An imbalance of synthesis and degradation causes overproduction of hyaluronan (HA) – a hallmark of joint swelling associated with RA caused by the undesired activation of three hyaluronan synthase genes: *HAS1*, *HAS2*, and *HAS3*, which seem to have distinct functions. IL1-β, TNF-α, and TNF-β strongly induce HA synthesis via NF-kappaβ signaling pathway – this pathway mediates hyaluronan synthase 2 (HAS2) mRNA expression, which modulates monocyte adhesion by CD44, initializing an inflammatory process wherein HA and adhesion molecules participate in the recruitment of immune cells [47]. TGF-β1 contributes most efficiently to accumulation of high-molecular-weight HA by upregulating HAS1/HAS2 expression and downregulating KIAA1199 expression in RA FLSs [48].

Calcineurin plays an important role in the T cell receptor-mediated signal transduction pathway. *CABIN1* encodes calcineurin-binding protein 1 – a negative regulator for calcineurin-mediated signal transduction in T lymphocytes. The abnormal activation of calcineurin by stimulation with IL-1β and TNF-α in RA synoviocytes may contribute to the pathogenesis of inflammatory arthritis; however, overexpression of CABIN1, a natural calcineurin antagonist, suppressed calcineurin activity by inhibiting IL-6 and MMP-2 production by rheumatoid synoviocytes [49]. Investigation of transgenic mice overexpressing hCABIN-1 demonstrated that *CABIN1* has an important role for in promoting FLS apoptosis, in attenuating cartilage and bone destruction and inflammation in RA [50].

GWAS approach has been successful in searching for susceptibility genes for multifactorial autoimmune diseases. Cumulative data from genome-wide JIA-focused screens indicate that the identified risk alleles are relatively common in general population, have only modest effects on risk, are located in intergenic regions and introns, and together explain only a small proportion of the variance in disease risk. The majority of identified JIA risk loci and variants represents genetic risk factors shared with other autoimmune diseases, pointing to a common genetic background with discrete biological pathways underlying autoinflammatory processes. Notably, several risk-contributing loci are not necessarily shared among different ethnic groups; thus, genetic risk factors for JIA likely differ among worldwide populations. The low prevalence and clinical heterogeneity of JIA have rendered the assembly of large cohorts difficult. We acknowledge that with the current sample size, this study was insufficiently powered to identify common variants having only modest effect sizes; therefore, we were unable to replicate genome-wide-significant associations found for several previously known JIA-predisposing genes, such as *PTPN22*, *STAT4*, *PTPN2*, *ANKRD55*, and *IL2*/*IL21* (4). Clearly, previous studies attempting to replicate RA-specific loci likely had limited power to detect associations with JIA. Large-scale GWASs of RA and JIA, respectively, render us prospects to identify potential common mechanisms in disease pathogenesis, which may lead to the identification of potential novel targets for therapeutic intervention and extending treatment strategies from commonly used molecular therapy with antibodies against TNF-α or IL-1 inhibitors to include recent advances in efforts to unravel effective targets influencing autoinflammatory and/or autoimmune processes.

Our findings markedly increase the estimated number of JIA susceptibility loci to be about 50. Their overlap with other autoimmune disease-related phenotypes infers shared etiologies for JIA and RA, giving an opportunity to identify therapies that more broadly influence JIA in the context of inflammatory arthritis risk. The implicated genes highlight the importance of autophagy in apoptosis resistance in inflammatory arthritis; DNA methylation complemented with the type I IFN signature network; and additional autoimmune and inflammatory pathways such as NF-κB, TGF-induced MAPK, CD200/CD200R1, Wnt, AKT, and ERK. Further functional characterization of essential pathways and pinpointing of genes through which their effects are mediated could provide additional insights into biological processes underlying the identified associations.

## Conclusions

The results of this study further emphasize the role of common genetic variation and add to the understanding of the genomic architecture influencing the risk of oligoarticular and RF-negative polyarticular JIA in Estonian patients. Certainly, more JIA-specific genetic risk factors remain to be identified, and well-powered GWASs are required for such identification. The future clinical relevance of our findings include contributions toward better understanding of JIA pathogenesis, identification of targets for drug development and/or repositioning of treatment, and presumably to improved prediction of JIA and its subtypes.

## Supplementary Information

Below is the link to the electronic supplementary material.

Supplementary file1 (DOCX 22 KB)

## Data Availability

The datasets generated and/or analyzed during the current study are not publicly available but are available from the corresponding author on reasonable request.

## Abbreviations

ANA: Anti-nuclear antigen
ILAR: International League of Associations for Rheumatology
JIA: Juvenile idiopathic arthritis
RA: Rheumatoid arthritis
RF: Rheumatoid factor
OA-JIA: Oligoarticular JIA
GWAS: Genome-wide association study
HLA: Human leukocyte antigen
MHC: Major histocompatibility complex
NK: Natural killer
OR: Odds ratio
SNP: Single-nucleotide polymorphism
SNV: Single-nucleotide variant
PCA: Principal component analysis
TNF: Tumor necrosis factor
IL: Interleukin
IFN: Interferon
Th: T helper
TGF: Transforming growth factor
FLS: Fibroblast-like synoviocytes
BMP: Bone morphogenetic protein
MMP: Matrix metalloproteinase
CCP: Cyclic citrullinated peptide
HA: Hyaluronan
