# Supplementary material for: Association analysis of juvenile idiopathic arthritis genetic susceptibility factors in Estonian patients
Source: Clin Rheumatol. 2021 Jun 8;40(10):4157–65. doi: 10.1007/s10067-021-05756-x (PMC8463396; doi:10.1007/s10067-021-05756-x)
Supplement: Supplementary file 1 — Supplementary file1 (DOCX 22 KB) [file 10067_2021_5756_MOESM1_ESM.docx]

**Supplementary Table 1. Descriptive laboratory data of the JIA study group (263 patients).**

| JIA subtype | No of ANA positive cases | No of HLA-B27 positive cases |
| --- | --- | --- |
| Oligoarthritis, persistent | 15 | 8 |
| Oligoarthritis, extended | 18 | 7 |
| Polyarthritis, RF negative | 7 | 6 |
| Polyarthritis, RF positive | 8 | - |
| Enthesitis-related arthritis | 7 | 2 |
| Psoriatic arthritis | 2 | - |
| Total No of positive cases / No of cases in whom the analyses was performed | 50 /252 | 23 / 102 |

Abbreviations: ANA – antinuclear antibody; HLA – human leukocyte antigen; RF – rheumatoid factor
